# Discursive constructions of family functions in forensic psychiatry: A critical ethnographic perspective

**DOI:** 10.1177/13634593231185263

**Published:** 2023-06-30

**Authors:** Jean-Laurent Domingue, Jean-Daniel Jacob

**Affiliations:** University of Ottawa, Canada; University of Ottawa, Canada

**Keywords:** critical theory, ethnography, family, forensic psychiatry, mental health

## Abstract

Significant barriers remain regarding the implementation of family-centred approaches in the domain of forensic psychiatry despite their effectiveness at increasing adherence to treatment, improving attendance to medical appointments, decreasing readmission rates and reducing episodes of relapse. We attribute these barriers to a fundamental gap in our understanding of the family function and its role within the forensic psychiatric system. Despite requesting to be included and considered as partners, some families feel excluded and sidelined, which causes distress, incomprehension and disengagement. We approached this tension at the discursive level through a critical ethnography of the Review Board and the work of Foucault on psychiatric power, which provided us with a unique opportunity to understand how the role of families are constructed and sustained in the Canadian forensic psychiatric system. To do so, we mobilized data stemming from ethnographic observations and documentary artifacts entitled ‘reasons for disposition’. Data analysis allowed us to identify two discursive constructions of familial functions: (1) families as repositories of information and (2) families as supervisory agents. These results have implications for health care professionals and administrators in forensic psychiatry who are increasingly adhering to family-centred care models without questioning what such care or what such family engagement entails.

The Canadian forensic psychiatric system is composed of health care, legal and social service institutions that interact with each other to preserve the safety of the public and provide mental health care to persons found unfit to stand trial (UST) or not criminally responsible on account of mental disorder (NCR). While the legislative framework guiding the system applies across the country, its implementation takes place at the provincial level. Each Canadian province puts in place Review Boards (RB), which are mental health tribunals that conduct hearings to determine whether persons UST/NCR represent ‘significant threats to the safety of the public’, and, if so, order conditions to preserve public safety (Criminal Code, *1985*: s.672.54).

In recent years, interest has been generated around the inclusion of families of persons UST/NCR in treatment decisions (Hörberg et al., 2015; Rowaert et al., 2016, 2017). Some scholars were interested in understanding the perspective of families on their relative’s offending, their mental illness and the mental health care they have received (Nordström et al., 2006). Others studied the supports, needs and interventions provided to the carers of offenders with mental illnesses (Absalom-Hornby et al., 2011; Absalom et al., 2010; Canning et al., 2009; MacInness, 2000; MacInnes and Watson, 2002; McCann et al.,1996; Rowaert et al., 2016; Tsang et al., 2002).

Family involvement in the planning and delivery of health care to individuals diagnosed with mental illnesses has been found to increase patients’ adherence to treatment plans, improve their attendance to medical appointments, decrease readmission rates and reduce episodes of relapse (Yeo Chen Kuan et al., 2018). For the Canadian forensic psychiatric system, where preserving the safety of the public is the paramount priority (Criminal Code, *1985*), these outcomes for patients have important social implications. Strong, reliable and prosocial relationships with family members have been found to reduce the risk of physical and psychological harm by persons UST/NCR (Bonta and Andrews, 2016; Douglas, 2014).

However, the implementation of family-involved mental health care models in the domain of forensic psychiatry has been met with resistance (Hörberg et al., 2015), leaving family members feeling disengaged from their relatives’ treatment teams (Rowaert et al., 2016). Rowaert et al. (2017) explained that family members had difficult and strenuous relationships with mental health professionals in part due to fact that information about their relative, and the forensic psychiatric system in general, was difficult to comprehend and hard to access. Concurrently, some mental health care professionals believed family engagement in the context of forensic psychiatry was not always in the patient’s or public’s interest because families were thought to impede the delivery of mental health care (Hörberg et al., 2015). Yet, families reported feeling powerless, socially isolated and alienated when their relationships with mental health professionals lack openness, confirmation and cooperation (Ewertzon et al., 2010).

Understanding the functions of families in forensic psychiatry is particularly important in a context where most of the research conducted in this domain stems from a desire to explore the relationship between families of persons UST/NCR and their health care team (Absalom-Hornby et al., 2011; Absalom et al., 2010; Canning et al., 2009; MacInness, 2000; MacInnes and Watson, 2002; McCann et al.,1996; Nordström et al., 2006; Rowaert et al., 2016; Tsang et al., 2002). Notwithstanding the clinical importance of recommendations made in these studies, including the need to provide families with information, to adopt a supportive approach and to include them in treatment decisions (Giacco et al., 2017; Javed & Herrman, 2017), the clinical emphasis placed on the relationship between families of persons UST/NCR and treatment teams has left many other interactions which exist between families and the broader forensic psychiatric system to be neglected and misunderstood. Indeed, the only study to have specifically looked at the experiences of families of persons UST/NCR in relation to the broader Canadian forensic psychiatric system amalgamated the experiences of families with those of persons UST/NCR and health/legal professionals (Livingston et al., 2016). While Livingston et al. (2016) were able to identify gaps regarding perceived procedural justice in the context of RB hearings, the experiences and perspectives of families remained diluted to the extent that they were presented alongside those of persons NCR, health professionals and legal professionals.

In the present article, we draw on the results of our study to illustrate the way the forensic psychiatric system discursively constructs the functions of families of persons UST/NCR. To do so, we first lay out the methodological foundations of the study. Then, we present the results and explore how, in forensic psychiatry, the functions of families transcend the clinical realm to the extent that families serve as essential repositories of information and as supervisory agents for forensic psychiatric hospitals. Finally, in our discussion, we suggest that these discursive constructions of family functions may be at the origin of tensions between mental health care professionals and families of persons UST/NCR and problematize the notion of family engagement in the context of forensic psychiatry.

## Methodological and theoretical foundations

A critical ethnography methodology was employed to investigate the identity construction of persons UST/NCR during RB hearings. Critical ethnography is used to study cultures that place individuals in a position of subjugation with the aim of identifying structures which allow for such a subjugation to take place (Soyini Madison, 2005; Thomas, 1993). Critical ethnographers use a variety of data sources to achieve such an aim (Hammersley and Atkinson, 2007). For this study, we conducted six interviews with nurses who worked in forensic psychiatry, observed 27 RB hearings totalizing 41 hours and collected data from a variety of documents, including 18 documents entitled ‘reasons for disposition’. Reasons for disposition are public documents produced by provincial RBs which provide the reasons why persons UST/NCR need disposition orders restricting their liberties for the public to be protected from them.

### Nurse interviews

The sampling of nurses was primarily guided by their willingness to participate. We recruited them through direct contact at team meetings and via email, with the authorization of the hospital administration. The following two inclusion criteria guided the selection of nurses: (a) having worked in forensic psychiatry; and (b) having assisted to at least one RB annual hearing. Informed consent was obtained prior to the commencement of the interviews. Given the small sample of participants, anonymity and confidentiality were particularly important ethical considerations. To protect anonymity, no demographic information was collected or reported, and participants were encouraged to select a location of their choice for the interview, thus preventing their colleagues from seeing them with the interviewer. Participants were also assigned an alphanumerical code (N01, N02. . .N06) and all identifying information was removed from their interview during transcription (i.e. names of cities and names of colleagues). Recording of interviews were deleted after transcription. Research Ethics Board approval for this study was obtained by the forensic psychiatric hospital where the nurse interviews took place (# 2019014) and from the first author’s academic institution (# H-07-19-4797).

### Observations of RB hearings

Observations of 27 RB hearings took place during a six-month period between June and November 2019. The RBs are composed of psychiatrists, psychologists, legal professionals and public members and decisions are made by majority vote. The RB hearings were open to the public, and they were held in a boardroom within the forensic psychiatric hospital. During these hearings, the person UST/NCR, their defense counsel, a Crown attorney and a representative of the forensic psychiatric hospital, which was usually the person UST/NCR’s treating psychiatrist, presented and discussed evidence in order to determine whether persons UST/NCR represent ‘significant threats to the safety of the public’, and, if so, determined the conditions necessary to preserve public safety (Criminal Code, *1985*: s.672.54). Despite being non-adversarial inquisitive hearings (Criminal Code, *1985*), the spatial distribution of participants during RB hearings and the processes inherent to them were reminiscent of criminal trials. A full description and analysis of the spatiotemporal particularities of the observed RB hearings are discussed elsewhere (Domingue et al., 2020, 2022).

### Reasons for Disposition

Reasons for disposition of four persons UST/NCR for whom we observed their RB hearing were accessed via the Lexis Advance Quicklaw legal database. This sample was legally and demographically representative of the population of persons UST/NCR. Creswell (2013) explained that samples for ethnographic studies should be representative of the entire cultural entity being studied. Contrary to positivist and postpositivist paradigmatic approaches, having a sample that was representative of the population was not important for generalizability purposes. Rather, having a sample that represented the Canadian UST/NCR population permitted for structures and discourses that sustained the construction of their identities to be thoroughly understood and problematized. Thus, we wanted our sample of reasons for disposition to include persons accused of different alleged index offences, including nonviolent, sexual and violent offences; persons of female and male genders; and persons of various ethnocultural backgrounds, including non-White, Indigenous and White.

### Data analysis

Analysis was completed using Norman Fairclough’s (1992) framework for conducting critical discourse analysis. Combining Fairclough’s (1992) critical discourse analysis method with a critical ethnography methodology was essential as it permitted a critical discursive analysis of social events, such as RB hearings, and an understanding of how discursive structure, such as the family, allowed for the forensic psychiatric culture to be sustained and (re)produced. It also allowed for us, as health care professionals in the domain of forensic psychiatry, to remain critical throughout the research process without feeling compelled to produce results that aligned with nursing disciplinary imperatives, which include, for example, family-centred and recovery-based models of care.

Fairclough’s (1992) analytical method comprises three steps. The first step is to understand why, how and by who the statements comprised within the data were produced, and why, how and by whom they were used. The second step is to look at the textual elements of the data to decipher its thematic content, the discursive materializations within it, and the semiotic structure of the text. The last step in Fairclough’s (1992) method is to question, using a theoretical framework, how and why the statements made within the text sustain, perpetuate and reproduce the hegemony of certain structures and discourses.

The theoretical framework was used as a starting point in our reflection about RB hearings and identity construction, and it combined the work of four scholars, namely Goffman (1961), Garfinkel (1956), Castel (1981) and Foucault (1975, 1999, 2003, 2004). Goffman (1961) and Garfinkel’s (1956) work allowed us to think about the RB hearing as a ceremony within the forensic psychiatric hospital that served two distinct interrelated roles: (1) maintaining the discipline of forensic psychiatry in a position of hegemony and (2) producing persons UST/NCR as dangerous individuals. The work of Castel (1981) and that of Foucault (1999, 2004) permitted us to think about how the concepts of risk and dangerousness intersected during RB hearings to legitimize restrictions imposed on the liberty of persons UST/NCR. A complete description of the theoretical framework is available in Domingue et al. (2020).

For the purpose of this article, we primarily lean on Foucault’s (2003) work related to psychiatric power and return to it in the discussion section. His work is particularly useful in the context of our study because it provides us with a conceptual understanding of how families, by sharing information about their relatives, sustain the hegemonic position of the forensic psychiatric discipline as the expert in the identification, treatment and reform of threatening persons living with mental illnesses.

## Results

It was evident, throughout our data collection, that beyond being supportive and active in the care and recovery of persons UST/NCR, families served functions aligned with the custodial impetus of the forensic psychiatric system. Out of our analysis, two of these functions became particularly apparent: (1) families as repositories of information and (2) families as supervisory agents. Indeed, families provided the forensic psychiatric system with information about their relatives’ prodromal signs of mental illnesses and historical information about their deviant conduct, which helped forensic psychiatric hospitals justify the person UST/NCR’s need for further detention or supervision. Concurrently, families served as supervisory agents for the forensic psychiatric system when their relatives entered or reintegrated the community. In the following paragraphs, we detail these two discursive constructions of family functions by mobilizing data stemming from our analysis of reasons for disposition and from observations of RB hearings.

### Families as repositories of information

In this section of the results, we illustrate, through the analysis of reasons for dispositions, how the forensic psychiatric system utilizes families to extract a maximum amount of information about persons UST/NCR, which is subsequently leveraged during RB hearings to justify their dangerousness and their need for supervision or detention. Our findings also highlight that through this instrumentalizing of families, highly confidential information about them, most likely disclosed in health care settings, is rendered public during the RB hearing and through the publication of the reasons for disposition.

Before a judge finds a person UST or NCR, fitness or criminal responsibility assessments are typically conducted by forensic psychiatrists. These assessments require the completion of biopsychosocial evaluations to situate the accused’s mental illness and their alleged crime in their life story. Often completed by social workers, the purpose of biopsychosocial evaluations is to provide historical information about an accused’s biological, psychological and social development. To achieve this task, social workers lean on many sources of information, including an interview with the accused, their previous medical records, and, importantly in the context of this article, interviews with the accused’s family members.

Once found UST or NCR, the information contained in the biopsychosocial assessment, including that provided by family members, is (re)mobilized in RB hearings and in reasons for disposition: “Background information was pulled from a biopsychosocial assessment dated [15 days before the UST finding]” (Reasons for Disposition 3). This use of biographical information in reasons for dispositions inserts the person UST/NCR’s clinical presentation in a family story characterized by deviancy. For example, in the case of a person found UST on various assault charges, information provided by their mother was used in their reasons for disposition to highlight a pattern of familial deviancy. Among other things, their biological father was presented as being an absent parent:

[The mother] was involved in a relationship with an individual named [name of mother’s boyfriend], for a period of 14 years. [The mother] explained that [her boyfriend] was a father figure to her son. [She] and [her boyfriend] separated in [year]. According to the mother, [the person UST] was relieved when she separated from [her boyfriend] as [the person UST] had heard rumours that [her boyfriend] was being unfaithful to her. (Reasons for Disposition 1)

The details about the person UST’s mother’s conjugal relationships allowed for the RB to problematize the lack of a stable father figures in their life. In doing so, the RB publicly exposed the full names of these father figures and produced statements about their alleged infidelity.

Beyond elements testifying to the stability of the family core during the person UST/NCR’s upbringing, families also served as repositories of historical information about their general conduct. By mobilizing this information in reasons for dispositions, RBs inserted alleged crimes and ongoing ‘abnormal’ conduct of persons UST/NCR in biographical patterns of behavioural and social deviancy. In the following excerpt, the person UST’s slow speech development, poor academic progress, emotional dysregulation and social ineptitudes, were recuperated by the RB as essential elements to include in the reasons for disposition:

According to [the person UST]’s mother, from Kindergarten to Grade 6, [the person UST] was not able to speak properly and had difficulty comprehending and remembering words. He had a difficult time understanding his school lessons and following course material. As a result, he began to fall behind in school. [The person UST]’s mother explained that he was assessed when in elementary school and found to be born with a “mental delay and mental retardation.” He was also diagnosed as being hyperactive. She stated that [the person UST] would leave the classroom if he was frustrated. He was eventually expelled at the age of 17, as a result of this behaviour. [The mother] also stated that her son had difficulty socializing with other students. (Reasons for Disposition 3)

By including information related to the life history of abnormal, but not necessarily dangerous, conduct in the reasons for disposition, the RB associates the alleged crime of persons UST/NCR and their current conduct in a lifelong pattern of abnormality and deviancy, thus legitimizing, in part, findings of dangerousness.

Concurrently and intertwiningly, information provided by family members of persons UST/NCR also allowed for RBs to situate their relative’s mental illness in a historical pattern of psychopathology. For example, in the following reasons for disposition, the RB used information reported by the mother of a person NCR to provide some perspective about the evolution of her son’s mental illness before the commission of his alleged crime:

His mother had stated that she has always been worried about [the person NCR]’s perceptions about his self-worth. She explained that he has always been too hard on himself, and is affected by things others say about him. She explained that he has suffered with depression and anxiety through different periods in his life. . . . The mother stated that [the person NCR] would also do “a lot of ruminating” on issues when he would become depressed after perceiving himself as a “loser.” She also believes that [the person NCR] has poor perceptions of himself, and has felt depressed over not moving forward in life. His mother stated that [the person NCR] also had poor body image, and that he lost a significant amount of weight toward the end of his High school years. . . Regarding a familial history of mental health, [the person NCR]’s mother said that she has suffered from periods of depression during her own life. She stated that one of her brothers has been diagnosed with Post Traumatic Stress Disorder [PTSD] with suicidal tendencies, and that she has another brother who has had a “manic episode” with “hallucinations.” (Reasons for Disposition 7)

Although the person NCR’s self-esteem and self-worth issues might have been significant when experienced, they only became relevant and important from a clinical and legal perspective because of the alleged offense. The person NCR’s self-esteem and self-worth issues were presented as being chronologically linked to his mental illness at the time of the index offence and, in some sense, to the crime itself. By including the information about the person NCR’s family history of mental illness in the reasons for disposition, the RB inserts the person’s mental illness and their crime in a hereditary pattern of familial psychiatric deviancy. Collaterally, the RB publicly exposes personal health information of family members used to establish such a pattern—the mother’s depression and the uncles’ PTSD, mania and hallucinations.

Such a public disclosure of familial personal health information was not an isolated case. In the following example of a person UST, it was information about their sibling, namely their diagnoses of dyslexia and attention deficit and hyperactivity disorder, that was rendered public through the publication of reasons for disposition:

[The person UST] is the eldest in a sib line of three. His sister is 20 years old and lives in [Canadian province]. She receives government financial support after being diagnosed with ADHD and dyslexia. He also has two younger half-siblings, a maternal half-sister, and a maternal half-brother. (Reasons for disposition 1)

The mother of the person UST also fell victim such a public disclosure of personal information. Her ‘transient’ nature and her financial need for social assistance was inscribed in her son’s reasons for disposition:

To date, [the person UST]’s mother has been unreachable. Further, [the psychiatrist] described her presence as only temporal, or short lived, in her son’s life. The Board heard from [the defence counsel] that his client’s mother is transient and supported by social assistance. However, [the person UST]’s mother does contact [the defence counsel] about once a week, usually by via email. She was present in Court the day her son was found unfit to stand trial. (Reasons for disposition 1)

The mere fact of having a sibling or a child found UST/NCR has the potential to place personal health and social information of individuals in the public domain. Yet, whether family members are aware that information they provide in the context of biopsychosocial assessments can be used in publicly accessible reasons for disposition is unknown.

### Families as supervisory agents

In this section of the results, we mobilize observations of RB hearings to demonstrate the discursive constructive of families as supervisory agents in the forensic psychiatric system. Our findings illustrate that when persons UST/NCR exit the forensic psychiatric hospital and reintegrate the community, families come to bear the supervisory responsibilities of the forensic psychiatric system to the extent that they are called to stringently observe their relative and report any deviant conduct back to the health care team.

Forensic psychiatric hospitals have the responsibility of detaining and supervising persons UST/NCR for whom provincial RBs ordered dispositions. When persons UST/NCR find themselves within the walls of the institution, supervision rests with the forensic psychiatric personnel, including nurses, and on the technologies of observation made available to them, which may include cameras and door passes.

However, in situations where persons UST/NCR leave the forensic psychiatric hospital (temporarily or permanently) and enter the community without being accompanied by hospital personnel, supervision becomes more difficult. Certain community-based housing options for persons UST/NCR replicate some of the supervisory features of the forensic psychiatric hospital:

The defence asked if the 24-hour supervised residence would have staff 24/day. The psychiatrist confirmed. The defence asked how big the residence was. . . . The defence asked if the residence was locked at night. The psychiatrist said he thought so, but that he wasn’t 100 percent certain, but confirmed that the doors are monitored. The defence asked if the patient would be required to submit an itinerary when he left the residence. The psychiatrist denied but said that residents are required to sign in and out at the residence. The defence asked if there was a curfew at the residence. The psychiatrist said yes. The defence asked if the hospital would be contacted if the patient didn’t return to the residence. The psychiatrist confirmed. (Observation 7)

In settings such as the one described above, the architectural elements of the hospital that permits close observation, including 24-hour supervision, locked doors, monitored entries and exits, supervised medication administration and curfews, extend beyond its walls insofar as they are replicated in community-based residences. If persons UST/NCR deviate from what is expected of them, the residence informs the hospital, thereby allowing the hospital to act and correct the deviation according to hospital procedures.

However, not all persons UST/NCR reintegrate the community via supervised residences. Some are discharged directly to independent living arrangements, while others reintegrate the community by residing with friends and family. In effect, some family members prefer for their relative reside with them as opposed to them having to stay in the hospital for a prolonged period or having to be discharged in a supervised group-home setting. In the following excerpt, the mother of a person NCR explained that she believed the hospital environment – a congregate living environment with a stringent surveillance structure – to be partly responsible for her son’s violent behaviours:

The chair speaking to the mother said: “We heard from the psychiatrist that your son isn’t ready to live in the community. What are your thoughts about that?” The mother explained that all the violent incidents took place while her son was in the hospital; none of them took place in the community. She then said: “All I can say is that environment plays a part in one’s behaviour. Home is a safe place.” She then explained that her son was heavily bullied as a child. She said that there is a lot of stress inside an institution. Hypothetically speaking she said: “I don’t know how I would manage in the system. I don’t know how any normal person can come out of this system normal.” (Observation 15)

In her plea to the RB, the mother of the person NCR contrasts the hospital setting to the family home environment. She describes the former as being stressful and conducive to violent incidents, and the latter as a safe environment exempt from bullying-related stress. While the mother may have stated that home was the best environment for her son, the psychiatrist seemed to think that the person NCR still needed the supervisory architecture of the hospital to manage his violent conduct.

During the same RB hearing, in an apparent attempt to convince the RB that the mother would be sufficiently equipped to supervise and manage her son in the community, the defence counsel asked the mother how she would supervise, ‘control’ and ‘manage’ her son’s problematic conduct, should he reside with her:

The defence asked the mother if she could control “drug use” within her house. The mother said yes. The defence asked how she would manage her son. She said she would have “behavioural contracts” and jobs. She further said that she had “contacts in the community.” The defence asked the mother if she had access to a car for her son to partake in the mandatory drug tests. The mom said yes. The defence asked the mother if she thought her son would comply. She said yes. (Observation 15)

For persons UST/NCR who reintegrate the community without transiting through supervised settings, supervision is achieved through the mobilization of family members. Indeed, in such circumstances, it is expected for their families to take on part of the hospital’s supervision responsibilities when persons UST/NCR reintegrate the community. In the excerpt above, these responsibilities related to the person NCR’s use of drugs and violent conduct. Surveillance duties thus extend outside the team of health care professionals who work in forensic psychiatry and permeate the intimacy of the relationship between a person UST/NCR and their family.

## Discussion

Our study highlighted the integral roles of families in the custodial function of the forensic psychiatric system. The results illustrated the way information given by families gets marshalled during RB hearings and in reasons for disposition to justify the dangerousness of persons UST/NCR. Further, they allowed us to understand how families come to bear supervisory functions when their relatives gradually reintegrate community settings. In the following paragraphs, we situate these results within relevant theoretical and empirical literature, we discuss practice implications for nurses and other health care professionals working in forensic psychiatry, and we present opportunities for further research.

### Anchoring dangerousness in a pattern of family deviancy

The most striking and ethically questionable problem our findings highlighted was the public divulgation of personal information belonging to family members of persons UST/NCR in the reasons for disposition. Among other things, this information related to family members’ medical diagnoses, romantic relationships and socioeconomical hardships. In the context of forensic psychiatry in Ontario (Canada) this is permitted by the articulation of two pieces of legislation, the Mental Health Act (MHA, 1990) and the *Criminal Code (1985)*. The latter stipulates that RB hearings are public in nature and the former establishes that personal health information can be disclosed to RBs by psychiatric hospitals without the consent of persons UST/NCR. This legal framework allows for the quasi-unregulated movement of personal health information from a confidential health care setting to a public legal setting (Domingue et al., 2023).

Although such a practice would be unthinkable, and even illegal, in other domains of health care (Personal Health Information Protection Act, 2004), in forensic psychiatry it serves an essential function; it inserts the dangerousness of persons UST/NCR in a pattern of intergenerational, intragenerational and biographical deviance (Foucault, 1999, 2003). Foucault (2003) explains that the interview gives structure to the immateriality of insanity. During the interview with the patient, the psychiatrist finds antecedents and collateral information about the families of persons living with mental illnesses to give somewhat of a body to the impalpability of mental illness. This search for antecedents situates mental illness within the pathological families of individuals and introduces the notion of heredity in psychiatry. Foucault (2003) explains that heredity establishes that specific illnesses can cause an illness of the same kind, or of any other kind, in descendants. This search for antecedents causes for a plethora of information to be assembled to situate and explain the mental illness and deviance of persons UST/NCR within their (pathological) families. In other words, by gathering and assembling information about families of persons UST/NCR, psychiatrists can establish a hereditary link between the social and medical deviancies of families and their descendants’ mental illnesses.

The inclusion of information about the psychiatric, behavioural and social deviancies of family members of persons UST/NCR in the reasons for disposition allows for the RB to purport that a person UST/NCR’s dangerousness originates not only from their own life course, but from a hereditary pattern of deviancy and potential for dangerousness. In effect, this association between the alleged crime, familial history and the life story of persons UST/NCR within the reasons for disposition not only establishes that persons UST/NCR are dangerous, but it also underscores that they have had the potential for dangerousness even before their birth (Foucault, 2003). Further, as Foucault (1975) explains, it is this association within a single document that legitimizes the detention/supervision of persons UST/NCR and the other disciplinary modalities meant to keep the public safe, such as urine toxicology screens:

“As the biography of the criminal duplicates in penal practice the analysis of circumstances used in gauging the crime, so one sees penal discourse and psychiatric discourse crossing each other’s frontiers; and there, at their point of junction, is formed the notion of ‘dangerous’ individual, which makes it possible to draw up a network of causality in terms of an entire biography and to present a verdict of punishment-correction” (p. 252).

The act of collecting information from families of persons UST/NCR through biopsychosocial assessments has effects which transcend the clinical realm as far as it provides forensic psychiatric hospitals and RBs with crucial evidence to justify the dangerousness of persons UST/NCR, to anchor this justification in a hereditary pattern of deviancy and to warrant the use of disciplinary measures to preserve so-called public safety.

Although it is unknown if families are aware that information provided about their relatives may be used in such a manner, we believe health care professionals who elicit this information have an ethical responsibility to inform family members of persons UST/NCR about this possibility. Sharing with families the potential ramifications associated with disclosing information about themselves and their relatives would allow them to decline participation in biopsychosocial assessments or to limit the content they decide to provide.

### Families and the Supervision of Relatives Beyond Hospital Walls

In addition to giving insight into how the information provided by families of persons UST/NCR serves to justify their relatives’ dangerousness and need for reform, our findings illustrate the integral role of families of persons UST/NCR in the supervisory function of the forensic psychiatric system. In effect, family members of persons UST/NCR were identified as bearing part of the supervisory role of the forensic psychiatric hospital in cases where their relatives are discharged from the hospital to family residences. They could be asked, for example, to supervise their relative’s use of drugs and to monitor their deviant conduct. By taking on these responsibilities, families become agents that ‘convey[ing] the norms of the state into the private sphere [of family life]’ (Donzelot, 1979: 58). This conveyance of norms from the State to the family allows for a local, efficient and cost-effective way of governing conduct (Paradis-Gagné and Holmes, 2021). In forensic psychiatry, this family-based governance constitutes a technology which permits for the supervisory and rehabilitation responsibilities of the hospital to extend beyond institutional walls and penetrate the private life-spheres of persons UST/NCR.

Many authors have identified that the multiple roles family members of persons living with mental illnesses must bear, such as that of ‘carer’ and that of ‘supervisor’, are sometimes conflicting and cause for internal conflicts and ambivalence towards their relative (Copeland and Heilemann, 2008; Kontio et al., 2017), particularly in cases where their relatives previously exhibited violent behaviours (Paradis-Gagné and Holmes, 2019). In complement to these authors who came to their conclusions by interviewing family members of persons living with mental illnesses, our results demonstrate that, in forensic psychiatry at least, these experiences may be rooted in a discursive construction of families as extensions to the disciplinary architecture of the forensic psychiatric hospital.

On that subject, Foucault (2003) explains that treatment and rehabilitation are only possible in psychiatric institutions because of architectural features of the environment in which it is dispensed, namely its disciplinary characteristics which allow for constant surveillance. He also noted that the surveillance architecture of psychiatric institutions is no different than that of other disciplinary institutions like barracks or prisons; it is organized in a hierarchical manner whereby a variety of employees, such as nurses, orderlies and guards, observe mentally ill patients, document their observations in the medical record and report information up to the psychiatrist (Foucault, 2003). When patients deviate from institutional expectations, they are subjected to interventions aimed at restricting their bodily movements and at correcting their conduct. The combination of such interventions with the supervisory structure of the psychiatric hospital produces the psychiatrist as (a) the omnipresent supervisor of the disciplinary institution and (b) the director of all disciplines (Foucault, 2003). However, our results have shown that the reach of psychiatry extends beyond the walls of the forensic psychiatric hospital to the extent that families of persons UST/NCR are instrumentalized as supervisory agents. In effect, families were portrayed as being responsible for the pervasive surveillance of their relative, for ensuring their relative attended scheduled urine toxicology screens, and most importantly, for relaying information about their relative back to the hospital.

This discursive construction of familial functions in forensic psychiatry raises serious practical and ethical questions for health care professionals and health care administrators. While many authors report benefits of involving families of persons UST/NCR in the provision of care (Park et al., 2018; Yeo Chen Kuan et al., 2018), they do not explain what this so-called ‘care’ entails. This becomes particularly problematic when the forensic psychiatric system seems to consider ‘care’ provided by families to be infused with disciplinary imperatives. In effect, this conceptualization of care is in stark contrast with the idea families have about participating in the care of their relative, which stems from a relational desire to support their relative navigate the hurdles inherent to the forensic psychiatric system, to be informed about this system and to accompany their relatives on their paths to recovery (Ewertzon et al., 2010; Giacco et al., 2017; Park et al., 2018; Rowaert et al., 2016, 2017). Therefore, before adhering to, sustaining and perpetuating the idea that family involvement in the care of persons UST/NCR should be the gold-standard of psychiatric treatment (Herrin et al., 2016; Igel and Lerner, 2016; Park et al., 2018), health care professionals and health care administrators in forensic psychiatry should clarify what it is they consider care to be and what it means for families to be involved in the provision of such care. In a context where family members feel powerless, socially isolated and alienated when relationships between them and mental health professionals lack openness, confirmation and cooperation (Ewertzon et al., 2010), this reflective work is crucial as it will increase transparency between families and health care teams and provide families with the opportunity to give an informed consent before participating in tasks being asked of them; tasks that may have unfavourable outcomes for their relatives’ social trajectories (i.e. supervision, discipline, the possibility of prolonged detention).

The results of our study highlight that there is a necessity to circumscribe what health care professionals mean when they refer to ‘family engagement’ and ‘family involvement’ in the care of persons UST/NCR. Understanding how this conceptualization of family involvement converges or diverges with the ambitions families who wish to be engaged in the care provided to their relative is also paramount. Thus, we must research the interactions between families and broader structures inherent to the forensic psychiatric system, such as lawyers, courts, review boards and social service organizations. This is essential to grasp the multifaceted consequences of involving families of persons UST/NCR in forensic psychiatric processes, to provide important social context to care teams who wish to engage families in treatment or safety-based decisions, and to guide broader policy and practice decisions related to client and family centred-care approaches in forensic psychiatry.

## Conclusion

In this article, we highlighted two of discursive constructions of family functions in forensic psychiatric that became apparent during a critical ethnography of the Ontario RB: (1) families as repositories of information and (2) families as supervisory agents. We subsequently problematized these constructions insofar as they may not coincide with the expectations families of persons UST/NCR have when they get involved in the care of their relative. These results have implications for health care professionals and health care administrators alike who are increasingly adhering to so-called family-centred care models without questioning what such care or what such family engagement/involvement entails.
